# Fifteen years of heroin-assisted treatment in a Swiss prison—a retrospective cohort study

**DOI:** 10.1186/s12954-020-00412-0

**Published:** 2020-10-13

**Authors:** Michael Liebrenz, Alex Gamma, Anna Buadze, Roman Schleifer, Stéphanie Baggio, Bruce Schwartz, Andres Schneeberger, Ambros Uchtenhagen

**Affiliations:** 1 Department of Forensic Psychiatry, Institute of Forensic Medicine, University of Bern, Falkenplatz 18, 3012 Bern, Switzerland; 2 Department of Psychiatry, Psychotherapy and Psychosomatics, Psychiatric Hospital, University of Zurich, Zurich, Switzerland; 3 Division of Prison Health, Geneva University Hospitals and University of Geneva, Geneva, Switzerland; 4 Department of Psychiatry and Behavioral Sciences, Montefiore Medical Center, Albert Einstein College of Medicine, New York, USA; 5 Psychiatrische Dienste Graubünden (PDGR), Chur, Switzerland; 6 Swiss Research Institute for Public Health and Addiction ISGF, University of Zurich, Zurich, Switzerland

**Keywords:** Heroin-assisted treatment, Prison, Jail, Incarceration, Opioid crisis, Opioid-using individuals, Social functioning

## Abstract

**Background:**

In the context of the current US opioid crisis and the compelling fact that a quarter to a third of all those addicted to heroin pass through its prisons and jails each year, the care of incarcerated opioid-using individuals (OUI) needs to be improved.

**Aims:**

Little has been published on the effectiveness or outcomes of heroin-assisted treatment (HAT), a treatment option for severely dependent OUI delivered in a prison setting. The aim of this study was therefore to evaluate such treatment since its implementation. The primary objective was to investigate whether heroin-assisted treatment was associated with severe detrimental health outcomes. The secondary objective was to compare the heroin-assisted treatment group with the general prison population in terms of occupational functioning.

**Design:**

Retrospective cohort study

**Setting:**

An open prison with 120 places

**Subjects:**

Data on 1885 male prisoners with a total of 2239 imprisonment periods between 2000 and 2015 was available. Ninety-seven inmates in heroin-assisted treatment were compared with 1788 inmates from the general prison population (reference group).

**Measurements:**

Mortality, medical complications (including overdoses), and work performance (days worked, sick days, and monthly wages earned).

**Findings:**

Inmates receiving HAT were on average 1 year younger (33.8 vs. 34.9 years), had longer prison stays (7.3 vs. 3.0 months), were more often of Swiss nationality (68.0% vs. 28.9%), and had committed more drug- and property-related offenses (49.5% vs. 23.2% and 63.9% vs. 38.3%, respectively) compared to the reference group. No serious heroin-related medical complication occurred during the 15-year window of observation among inmates with heroin-assisted treatment. Their work performance was comparable to that of the reference group.

**Conclusions:**

This study shows that heroin-assisted treatment can be a valuable treatment option for severely dependent OUI during imprisonment, can be delivered safely by prison health staff over extended periods of time, and allows OUI in treatment to achieve work performance rates comparable to that of the general prison population.

## Introduction

In the context of the current opioid crisis in the USA, and against the backdrop that a quarter to a third of all US residents addicted to heroin pass through US prisons and jails each year [1], care for incarcerated opioid-using individuals (OUI) has become a focus of scientific interest. A smarter war on drugs [2], with addiction and overdose initiatives that address the current opioid epidemic and particularly envisage a criminal justice-based continuum of care for OUI, has been called for [3]. This implies a need for studies that not only evaluate medications used to treat opioid use disorders (OUD) in certain populations, but also develop better ways of assessing the outcomes of relevant therapeutic interventions [4].

Similar considerations, demands, and research questions arose some time ago in Switzerland, a federally governed European country. At the height of its heroin epidemic in the late 1980s, Switzerland had an estimated 30,000–40,000 OUI, i.e., 0.5–0.8% of the total population [5]. The drug policy response to this crisis was inconsistent, fluctuating between liberal and repressive approaches. After conditions had deteriorated to the point where open drug scenes existed in several Swiss cities, a national harm-reduction policy was adopted in 1991. It formally permitted the implementation of low-threshold medication-assisted treatment (MAT) with methadone and (later) buprenorphine, needle and syringe exchange services, and supervised consumption rooms [6]. Severely dependent OUI over the age of 18, who had failed MAT (defined as using additional, illegally obtained opioids despite receiving prescription medication) at least twice, were eligible to receive heroin-assisted treatment (HAT). Permission and control mechanisms were put into effect by the Federal Office of Public Health [7].

Under HAT, illegal “street heroin” is replaced by medically prescribed, pharmaceutically pure diacetylmorphine. HAT is more strictly regulated than MAT and does not allow take-home administration. Thus, HAT restricts OUI in everyday life more than MAT does and represents a therapeutic last resort for a seriously ill subpopulation. This is also reflected in the distribution of treatment frequencies: Of the approx. 19,400 OUI in treatment today, over 90% receive MAT, while only 1752 or about 8% receive HAT with diacetylmorphine [8].

In Switzerland, HAT was first evaluated as a treatment alternative in pilot trials in the 1990s [9]. The positive findings were later confirmed in other countries as well [10–12].

Since 1999, HAT has been introduced as a limited medical application in specialized outpatient clinics [13, 14]. In 2008, harm reduction and heroin prescription were legally anchored in the narcotics law [15]. Since a large number of OUI were coming into contact with the criminal justice system via drug-related crime and/or violations of the narcotics law, a paradigm shift occurred in the 1990s, enabling the start and continuation of MAT during detention in jail and/or prison. In addition, two Swiss prisons (Schöngrün penitentiary in 1995 and Realta penitentiary in 2000/2001) also made it possible for inmates to start and continue HAT. After the closure of the former in 2015 [16], there is, to the authors’ knowledge, only one correctional facility worldwide that offers HAT to severely dependent inmates during incarceration.

In view of the extreme paucity of data on the outcomes associated with HAT when delivered in prison settings, the aim of this study was to evaluate HAT since its implementation in the Realta prison. The main objective of the study was to investigate whether HAT was associated with detrimental health outcomes, including death, overdose, and medical complications. The secondary objective was to compare the HAT group with the general prison population (reference group) in terms of occupational functioning. The findings of this study should thus serve to provide a better understanding of HAT use in prison settings, identify benefits and risks associated with the use of HAT in a prison population, and inform clinical practice on the functioning level of OUI using HAT during periods of imprisonment.

## Materials and methods

### Study design and setting

This was a retrospective cohort study including inmates from the Realta prison, Switzerland, who either received HAT or did not (i.e., inmates from the general prison population serving as the reference group, labeled “non-HAT” below). It is an open prison with 120 places, of which six were treatment slots for HAT in 2000; there are 10 slots today. Both MAT and HAT are available to male OUI who have been sentenced to imprisonment. Treatment can either be continued on the basis of a medical referral or initiated during the prison term. Heroin is delivered twice daily between 7:00 and 7:15 a.m. and 6:30 and 6:45 p.m. by two nurses. The detainees administer the heroin themselves either intravenously, intramuscularly, or orally in a dispensing room (pictures available), where they are monitored from the adjoining room.

Work is a mandatory part of imprisonment in Realta. Most jobs are assigned to inmates by staff, with the exception of work on weekends (e.g., in the kitchen or in the fields), which is done on a volunteer basis. Also, barn work is considered more demanding (requiring more physical strength as well as getting up early) than other kinds of work and is preferentially assigned to more motivated or capable inmates.

### Subjects

In 2016, data on all inmates imprisoned between 2000 and 2015 were extracted from the electronic prison database. One hundred eleven prisoners had received HAT during the study period. Most inmates had only one prison term during the observation period, but some had several terms (Table 1). Subjects with several prison terms were allocated to the HAT group if they had received HAT during at least one term. It turned out, however, that all HAT participants with several terms had received HAT in every single one of their terms. About half (46.4%) of the subjects receiving HAT additionally received methadone, and in 4 (4.1%) cases, HAT was stopped and replaced with morphine or buprenorphine. Individuals receiving only MAT were excluded from the final sample because of the small sample size (*n* = 14) and are thus not part of the final HAT group. The final number of subjects in the HAT group was therefore *n* = 97. All non-HAT reference subjects (*n* = 1885) were included in the study. The non-HAT group did not include individuals who had participated in MAT during the observation period. Both groups were already naturally matched on sex and age, and further matching was not attempted.

**Table 1:** Basic characteristics of prison inmates

	Total	Non-HAT	HAT^a^	Missing, *N* (%)	*p*^b^	*p*_Bonf_^c^
*N* inmates (%)	1885 (100)	1788 (94.9)	97 (5.2)	0 (0)	n.a.	n.a.
Prison terms						
• *N*	1591 inmates: 1 term 228 inmates: 2 terms 52 inmates: 3 terms 10 inmates: 4 terms 3 inmates: 5 terms 1 inmate: 6 terms Total: 2239 terms	1529 inmates: 1 term 204 inmates: 2 terms 43 inmates: 3 terms 8 inmates: 4 terms 3 inmates: 5 terms 1 inmates: 6 terms Total: 2119 terms	62 inmates: 1 term 24 inmates: 2 terms 9 dinmates: 3 terms 2 inmates: 4 terms Total: 120 terms	0 (0)	n.a.	n.a.
• Total length [months], median (IQR, min–max)	3.1 (5.39, 0.0–101.0)	3.0 (4.94, 0.0–101.0)	7.3 (10.71, 0.3–40.9)	0 (0)	0.000	n.a.
Sex, *N* (%), male	1885 (100)	1788 (100)	97 (100)	0 (0)	n.a.	n.a.
Age, mean (SD, min–max)^d^	33.8 (10.83, 17.0–85.0)	33.8 (11.01, 17.0–85.0)	34.9 (6.61, 22.0–52.0)	0 (0)	0.000	n.a.
Nationality, *N* (%), Swiss	583 (30.9)	517 (28.9)	66 (68.0)	349 (15.6)	0.000	n.a.
Index offense, *N* (%), with ≥ 1 offense				152 (6.8)		
• Drug	462 (24.5)	414 (23.2)	48 (49.5)		0.000	0.000
• Traffic, light^e^	216 (11.5)	205 (11.5)	11 (11.3)		0.97	1.0
• Traffic, severe^f^	100 (5.3)	99 (5.5)	1 (1.0)		0.05	0.9
• Arson	7 (0.4)	7 (0.4)	0 (0.0)		0.54	1.0
• Kidnapping	3 (0.2)	3 (0.2)	0 (0.0)		0.69	1.0
• Sex	41 (2.2)	40 (2.2)	1 (1.0)		0.43	1.0
• Homicide/manslaughter	21 (1.1)	1 (1.1)	1 (1.0)		0.94	1.0
• Property	747 (39.6)	685 (38.3)	62 (63.9)		0.000	0.000
° Fraud	122 (6.5)	115 (6.4)	7 (7.2)		0.76	1.0
° Theft	597 (31.7)	541 (30.3)	56 (57.7)		0.000	0.000
° Robbery/extortion	75 (4.0)	65 (3.6)	10 (10.3)		0.001	0.018
° Other^g^	108 (5.7)	100 (5.6)	8 (8.2)		0.27	1.0
• Assault	173 (9.2)	162 (9.1)	11 (11.3)		0.45	1.0
• Weapons^h^	24 (1.3)	21 (1.2)	3 (3.1)		0.10	1.0
• Obstruction of justice^i^	82 (4.4)	75 (4.2)	7 (7.2)		0.16	1.0
• Other^j^	752 (39.9)	730 (40.8)	22 (22.7)		0.000	0.000
• Total violent crimes^k^	289 (15.3)	270 (15.1)	19 (19.6)		0.23	1.0
• Grand total	1674 (88.8^l^)	1586 (88.7^l^)	88 (90.7^l^)		0.54	1.0

*HAT* heroin-assisted treatment, *IQR* interquartile range, *min* minimum, *max* maximum, *N* number, *SD* standard deviation

^a^Includes heroin with or without other substituents (46.4% persons with methadone and 4.1% with morphine or buprenorphine)

^b^Kruskal-Wallis test for period length, *t* test for age, Pearson’s chi-squared test for frequencies otherwise

^c^Bonferroni-corrected *p* value correction only applied to offense-related variables

^d^Age is at the middle of an inmate’s imprisonment period. In the case of several stays, the mean is taken

^e^Includes driving without a license and violations of traffic and transportation laws

^f^Includes drunk driving and hit-and-run

^g^Includes handling stolen goods and computer fraud

^h^Includes any violation of weapons laws

^i^Includes violence or threats against public authorities or officials, contempt of official orders, prevention of an official action

^j^Includes property damage, violations of immigration laws, refusal to present personal identification, libel/slander, violations of animal protection laws, panhandling, and others

^k^Includes homicide, manslaughter, assault, sex offenses, robbery, and blackmail

^l^The numbers do not sum to 100% due to missing values

The eligibility criteria for HAT during detention are the following:

- At least 2 years of demonstrable heroin addiction
- At least two failed recognized treatment attempts (such as a MAT)
- Medical, psychological, or social deficits due to opioid drug use
- Consent of the referring legal authority (probation and correctional services)

### Measures

#### Medical information

In the HAT group, we collected information on mortality, overdoses, and other medical complications (health and safety issues during and after heroin distribution) from prison medical records. In accordance with previous studies, non-fatal heroin overdose was defined as any of the following symptoms: suppressed breathing, turning blue, collapsing, losing consciousness, and being unable to be roused [17, 18].

#### Occupational functioning

Work performance was documented by prison staff using six variables: number of days worked, number of days sick, number of weekend days worked, number of days of barn work, salary (all per month), and occupational accidents.

#### Control variables

Other variables recorded by prison staff included age, sex, nationality, dates of admission and discharge, use of psychiatric services, and index offense.

### Statistical analysis

Analyses were performed on the subject level, aggregating, if necessary, over multiple prison terms. Aggregation consisted of summing frequencies of categorical variables over all of a subject’s prison terms and by summing continuous variables and computing per-month averages based on the total length of a subject’s incarceration.

Between-group comparisons of frequencies used chi-squared tests, while Kruskal-Wallis tests were applied to continuous variables due to their mostly non-normal distributions. Bonferroni correction for multiple comparison was applied to sets of variables related to a common subject area. For the outcomes of secondary interest, multivariable analyses were run. For variables showing a normal distribution (disregarding the excess zeros), we used a linear model; for those showing Poisson-like distributions, we used an exponential model. Predictors included group (HAT or non-HAT), age, Swiss nationality, use of psychiatric services, length of prison term, and the date of the mid-point of the prison term. An excess of zeros was present for all variables, prompting the use of hurdle models [19–21]. The analysis was conducted on the level of subjects. However, robust standard errors were calculated to account for the clustering of prison terms within subjects [21–26]. Models were implemented using Stata’s churdle command.

Missing values were not imputed. There were no missing values for the primary outcomes and 0.1% for the secondary outcome. Percentages of missing values were higher for nationality (15.6%) and index offenses (6.8%), but there were no missing values for the use of psychiatric services. Results of a power analysis for the chi-squared and Kruskal-Wallis tests are available in the supplement. R version 3.4.2 and Stata version 14.2 were used for analysis.

## Results

### Preliminary analyses

In total, there were 1885 inmates, 97 (5.2%) HAT and 1788 (94.9%) non-HAT. The mean age was 33.8 years, with about a year’s difference between the groups. Swiss was by far the most frequent nationality in the two groups. HAT subjects were significantly more likely to be Swiss (68.0%) than non-HAT subjects (28.9%). The median length of imprisonment was 3.1 months and ranged from a few days to over 8 years. Compared to subjects receiving HAT, non-HAT subjects had shorter stays (3.0. vs. 7.3 months; Table 1).

Apart from the unspecific category “other,” the most frequent reason for imprisonment was a property-related offense (≥ 35 % in both groups), of which theft was the most common (> 35% of all property-related offenses). A drug-related offense was the second most frequent reason for imprisonment (> 20% in both groups). Assault had a 10% frequency in both groups, while violent crimes made up 15–20% of all offenses. Compared to non-HAT inmates, HAT inmates had committed statistically significantly more drug-related offenses (49.5% vs. 23.2%), property-related offenses (63.9% vs. 38.3%, with theft also being more frequent at 57.7% vs. 30.3%), and robbery and extortion offenses (10.3% vs. 3.6%). They were less often found to have committed severe traffic offenses (1.0% vs. 5.5%) and “other” crimes (22.7% vs. 40.8%; Table 1).

Of HAT subjects, 22.7% had at least one consultation with the consiliary psychiatric service, whereas only 1.5% of the reference group subjects did.

### Primary objective: mortality, overdoses, and other medical complications in the HAT group

No opioid overdose death, no non-fatal overdose, and no serious medical complications were observed in the HAT group at any time. In one case, a HAT subject showed a “slight clouding of consciousness for 10 min after drug administration,” but this needed no further medical intervention and no treatment with naloxone hydrochloride, for example. No overdose-related morbidity was observed including rhabdomyolysis, pulmonary symptoms (e.g., pneumonia, edema), cardiac arrhythmia, seizures, and paralysis. No safety issue during or after heroin distribution was ever reported.

### Secondary objective: occupational functioning

Inmates worked for a median of 18.6 days per month and had a median of zero sick days. The median number of work days was slightly higher (18.7 vs. 17.0), and the number of sick days lower (0.0 vs. 1.0) in the non-HAT group. HAT subjects, however, had more days of barn work (both medians zero, means 3.4 vs. 1.9) and a higher salary (36.6 CHF vs. 33.0 CHF). To be maximally informative, Table 2 shows the results broken down by categories of days. eTable 1 in the supplement shows the results in terms of medians.

**Table 2:** Work performance per month of imprisonment

	Total	Non-HAT	HAT^a^	Missing, *N* (%)	*p*^b^	*p*_Bonf_^c^
*N* (%)	1885 (100)	1788 (94.9)	97 (5.2)	0 (0)	n.a.	
Salary [Swiss francs], median (IQR, min–max)	33.5 (13.28, 0.0–578.3)	33.0 (13.36, 0.0–578.3)	36.6 (12.04, 0.0–72.2)	3 (0.13)	0.001	0.005
*N* work days	*N* (%)	*N* (%)	*N* (%)	3 (0.13)	0.0005	0.0025
0	170 (9.0)	165 (9.2)	5 (5.2)			
1–4	57 (3.0)	47 (2.6)	10 (10.3)			
5–9	137 (7.3)	118 (6.6)	19 (19.6)			
10–14	196 (10.4)	186 (10.4)	10 (10.3)			
15–19	715 (38.0)	685 (38.4)	30 (30.9)			
20–24	562 (29.9)	539 (30.2)	23 (23.7)			
25+	45 (2.4)	45 (2.5)	0 (0.0)			
*N* sick days	*N* (%)	*N* (%)	*N* (%)	3 (0.13)	0.0005	0.0025
0	1403 (74.6)	1354 (75.9)	49 (50.5)			
1–4	316 (16.8)	285 (16.0)	31 (32.0)			
5–9	86 (4.6)	76 (4.3)	10 (10.3)			
10–14	38 (2.0)	33 (1.9)	5 (5.2)			
15–19	29 (1.5)	27 (1.5)	2 (2.1)			
20+	10 (0.5)	10 (0.6)	0 (0.0)			
*N* weekend days at work	*N* (%)	*N* (%)	*N* (%)	3 (0.13)	0.03	0.15
0	1521 (80.8)	1437 (80.5)	84 (86.6)			
1–4	207 (11.0)	195 (10.9)	12 (12.4)			
5+	154 (8.2)	153 (8.6)	1 (1.0)			
*N* days barn work	*N* (%)	*N* (%)	*N* (%)	3 (0.13)	0.001	0.005
0	1628 (86.5)	1556 (87.2)	72 (74.2)			
1–4	44 (2.3)	37 (2.1)	7 (7.2)			
5–9	37 (2.0)	34 (1.9)	3 (3.1)			
10–14	31 (1.6)	30 (1.7)	1 (1.0)			
15–19	61 (3.2)	54 (3.0)	7 (7.2)			
20+	81 (4.3)	74 (4.2)	7 (7.2)			

*HAT* heroin-assisted treatment, *IQR* interquartile range, *max* maximum, *min* minimum, *N* number

^a^Includes heroin with or without other substituents (46.4% persons with methadone and 4.1% with morphine or buprenorphine)

^b^Kruskal-Wallis test for salary, otherwise chi-squared test

^c^Bonferroni-corrected *p* values

Multivariable analyses revealed only a few effects that were of both practical and statistical significance: HAT was associated with about one and a half fewer work days, about one and a half more sick days, and about 1 day less of weekend work. Being of Swiss nationality was associated with about half a day less of being sick and about one additional day of barn work. Five additional months of imprisonment were associated with one additional work day (eTable 2 in the Supplement).

No occupational accidents associated with participation in HAT were recorded during the observation period.

Missingness on nationality and index offense had a number of sizeable and statistically significant effects on work-related outcomes and other variables, notably on the monthly number of work days, weekend days at work, monthly salary, and use of psychiatric services (eTables 3 & 4 in the Supplement).

## Discussion

Fifteen years after the introduction of heroin-assisted treatment for incarcerated opioid-using individuals in a Swiss open prison, we found no evidence for increased mortality, overdoses, or severe medical complications. In fact, over the time period evaluated, no drug-related death occurred in inmates receiving HAT.

Here, it should be emphasized that this was an open prison setting, i.e., inmates are given the opportunity to leave the prison during the day and work offsite, thus increasing their likelihood of being exposed to illegal street opioids. Various studies have shown that the mortality rate of OUI immediately after release from closed prison without MAT is increased and remains at an elevated level [27–29]. The elevated risk of fatal overdoses in this population has been linked to the loss of tolerance for opioids during incarceration, in combination with a lack of psychoeducation about the consequences of resumption of opioid use in unchanged dosages [30, 31]. In contrast, the introduction and continuation of MAT are associated with a reduction in mortality after release from prison during community re-entry [32, 33]. Our findings in an open prison complement these reports and underline that this protection holds true even for severely dependent OUI with HAT, who were considered unresponsive to MAT.

On measures of work performance, which can be understood as a proxy for occupational and social functioning [34–36], we found comparable levels of days worked, sick days, and monthly salary between OUI in treatment with HAT and the general prison population. This finding is surprising for three reasons. First, inmates receiving HAT represent a group of severely dependent OUI who have failed other forms of treatment, including MAT. Significant impairments in social and work performance for severely addicted individuals are well documented elsewhere [37–39]. Second, inmates who received HAT seemed to suffer to a significantly higher degree from co-morbid and impairing mental disorders, as reflected by the fact that they were referred to or sought out psychiatric consultation 15 times more often than non-HAT inmates. Mental illness with a co-morbid substance use disorder is also known to decrease levels of work performance [40]. Third, HAT-receiving inmates suffered to a greater extent from physical illnesses and infectious diseases, as indicated by the higher frequency of pain, gastrointestinal, and antiviral medications prescribed. Periods of physical illness are also associated with an increased risk of inability to work [41].

It was equally surprising that no occupational accidents associated with participation in HAT were reported during the observation period. From the perspective of legal authorities, next to fears about diversion of substances (stealing and smuggling drugs), the risk of accidental overdose and concerns over problems with the operation of machinery had been major obstacles to the introduction of MAT or HAT in Swiss prisons.

Due to the closure of the Schöngrün penitentiary in 2015, we could not evaluate its HAT program. However, after the introduction of HAT in conjunction with other harm-reduction approaches (e.g., needle exchange programs for injecting OUI) in Schöngrün, the number of fatal and non-fatal heroin overdoses among incarcerated OUI fell dramatically over a period of 9 years [42].

A recent meta-analysis of randomized trials of HAT for refractory heroin addiction among non-incarcerated OUI found that “heroin prescribing, as part of the highly regulated regimen, is a feasible and effective treatment for a particularly difficult-to-treat group of heroin-dependent patients who have not responded to standard treatments such as oral methadone maintenance or residential rehabilitation” [43]. The meta-analysis focused on treatment retention, illegal heroin use, mortality, and side effects and found improvements in mental and physical health as well as in social functioning [44]. Our observational study underscores these findings and provides evidence that these results also hold true for HAT administered to OUI under conditions of imprisonment. Additionally, we are able to report that the work performance and earnings of prisoners in a HAT program were comparable to the general prison population. This finding is a further indication of HAT’s usefulness in terms of improvements in the functioning and rehabilitation of affected individuals.

Brinkley-Rubinstein et al. [3] correctly point out that there is great heterogeneity within the prison system; thus, “what is possible in a rural (…) county will not always be comparable to what can be implemented in some big urban environments,” and thus, our findings from Switzerland might be of less relevance to the USA, which would constitute an important limitation. However, our data—in conjunction with the existing literature—provides three arguments for the introduction of a “criminal justice continuum for opioid users”: first, it shows that there are treatment options available even for severely dependent OUI and that those forms of treatment do not lead to increased mortality during periods of incarceration; second, those treatments can be delivered safely by prison health staff over extended periods of time; third, OUI in treatment achieve a work performance comparable to the general prison population. The last economic argument might prove to be of relevance in a correctional system which attaches great importance to the work activities of detainees [45].

### Limitations

Our study has important limitations. Sample sizes, especially the relatively small group of inmates enrolled in HAT, limited detectable effects (see power analysis in supplement). This limitation is inherent, as the HAT group actually constitutes the entire current population of heroin-treated prisoners in Switzerland. There were some sizeable differences in work performance and other variables between subjects with and without missing data on nationality and index offenses. This means that the multivariable associations (reported in the supplement) between work performance on one hand, and nationality and index offense on the other, are less reliable. The main analyses that compare outcomes between HAT subjects and reference subjects are, however, unaffected.

## Conclusions

This study shows that heroin-assisted treatment can be a valuable treatment option for severely dependent opioid-using individuals during periods of imprisonment. It also shows that HAT can be delivered safely by prison health staff over extended periods of time and that opioid-using individuals in such a treatment program achieve work performance comparable to that of the general prison population.

## Supplementary information

**Additional file 1.** Supplementary tables.

## Data Availability

The datasets used and/or analyzed during the current study are available from the corresponding author on reasonable request.
