# Supplementary material for: Fifteen years of heroin-assisted treatment in a Swiss prison—a retrospective cohort study
Source: Harm Reduct J. 2020 Oct 13;17:67. doi: 10.1186/s12954-020-00412-0 (PMC7552491; doi:10.1186/s12954-020-00412-0)
Supplement: Supplementary file 1 — Additional file 1. Supplementary tables. [file 12954_2020_412_MOESM1_ESM.docx]

**Supplement to manuscript "Fifteen years of heroin-assisted treatment in a Swiss prison"**

Table of contents:

eTable 1. Work performance per month of imprisonment (median (IQR, min – max)) – p. 2

eTable 2. Multivariable models of monthly work performance - p. 2

eTable 3. Effects of missing data on nationality on outcomes and predictors - p. 3

eTable 4. Effects of missing data on index offense on outcomes and predictors - p. 4

Power analysis – p. 5

**eTable 1. Work performance per month of imprisonment (median (IQR, min – max))**

|  | Total | Non-HAT | HAT^a^ | p^b^ | p_Bonf_^c^ |
| --- | --- | --- | --- | --- | --- |
| N | 1885 | 1788 | 97 | n.a. |  |
| N work days | 18.6 (7.45, 0.0 - 34.5) | 18.7 (6.94, 0.0 - 34.5) | 17.0 (12.17, 0.0 - 24.7) | 0.003 | 0.15 |
| N sick days | 0.0 (1.03, 0.0 - 30.4) | 0.0 (0.92, 0.0 - 30.4) | 1.0 (2.98, 0.0 - 15.4) | 0.000 | 0.000 |
| N weekend days at work | 0.0 (0.27, 0.0 - 12.2) | 0.0 (0.32, 0.0 - 12.2) | 0.0 (0.02, 0.0 - 6.6) | 0.26 | 1.0 |
| N days barn work | 0.0 (0.00, 0.0 - 27.8) | 0.0 (0.00, 0.0 - 27.8) | 0.0 (1.13, 0.0 - 22.6) | 0.044 | 0.22 |
| Salary [Swiss Francs] | 33.5 (13.28, 0.0 - 578.3) | 33.0 (13.36, 0.0 - 578.3) | 36.6 (12.04, 0.0 - 72.2) | 0.001 | 0.005 |

^a^includes Heroin with or without other substituents (46.4% persons with Methadon and 4.1% with morphine or buprenorphine)

^b^Kruskal-Wallis test

^c^Bonferroni-corrected p-values

HAT = Heroin-assisted treatment, IQR = Interquartile Range, max = maxiumum, min = minimum, N = number

**eTable 2. Multivariable models of monthly work performance**

|  | N work days | N days sick | N weekend days worked | N days barn work | Salary |
| --- | --- | --- | --- | --- | --- |
| Model type | Linear hurdle | Exponential hurdle | Exponential hurdle | Linear hurdle | Linear hurdle |
| Coefficient type | Linear | Linear | Linear | Linear | Linear |
|  |  |  |  |  |  |
| HAT | -1.62* [-3.09 - -0.15] | 1.73** [0.46 – 3.00] | -0.81*** [-1.25 - -0.37] | 0.79 [-0.51 -2.09] | 0.79 [-2.79 - 4.37] |
| Swiss nationality | n.a.^a^ | 0.45* [0.05 - 0.85] | -0.19 [-0.59 - 0.22] | 0.82* [0.17 -1.47] | 2.18 [-0.66 – 5.02] |
| Age | -0.04** [-0.07 - -0.01] | 0.02* [0.00 - 0.04] | 0.01 [-0.01 - 0.03] | -0.04** [-0.07 - -0.01] | 0.01 [-0.16 - 0.17] |
| N index offenses | 0.13 [-0.03 - 0.28] | 0.06+ [-0.00 - 0.12] | -0.05 [-0.12 - 0.01] | -0.01 [-0.10 - 0.08] | -0.10 [-0.63 - 0.43] |
| Length of prison term [months] | 0.20** [0.06 - 0.35] | -0.05** [-0.09 - -0.01] | 0.02 [-0.01 - 0.06] | 0.04 [-0.01 - 0.08] | 0.34 [-0.25 - 0.93] |
| Had psychiatric consultation | -0.31 [-2.84 - 2.21] | 0.03 [-1.17 - 1.24] | -0.49 [-1.54 - 0.57] | 1.56 [-0.85 – 3.97] | 2.87 [-2.17 – 7.92] |
| Date of imprisonment (mid-point) | 0.00*** [0.00 - 0.00] | -0.00*** [-0.00 - -0.00] | 0.00* [0.00 - 0.00] | 0.00* [0.00 - 0.00] | 0.00* [0.00 - 0.00] |

^a^Model did not converge unless this variable was omitted

+ p<0.10, * p<0.05, ** p<0.01, *** p<0.001

HAT = Heroin-assisted treatment , N = Number

**eTable 3.** **Effects of missing data on nationality on outcomes and predictors**

|  | Nationality non-missing (N=1920^a^) | | | | Nationality missing (N=359^a^) | | | |  |  |
| --- | --- | --- | --- | --- | --- | --- | --- | --- | --- | --- |
|  | Mean | SD | Median | IQR | Mean | SD | Median | IQR | Effect size^b^ | P-value^c^ |
|  |  |  |  |  |  |  |  |  |  |  |
| N work days | 16.34 | 7.01 | 18.84 | 6.76 | 12.46 | 8.57 | 15.60 | 16.78 | 0.52 | 0.0005 |
| N sick days | 1.57 | 3.84 | 0.00 | 1.01 | 1.11 | 2.77 | 0.00 | 0.80 | 0.12 | 0.48 |
| N weekend days at work | 0.90 | 2.03 | 0.00 | 0.20 | 0.59 | 1.67 | 0.00 | 0.00 | 0.16 | 0.04 |
| N weekend days at work | 1.94 | 5.78 | 0.00 | 0.00 | 1.86 | 5.52 | 0.00 | 0.00 | 0.01 | 1.00 |
| Salary | 35.42 | 23.17 | 33.69 | 13.86 | 29.02 | 23.38 | 30.85 | 25.10 | 0.27 | 0.0005 |
| Age | 33.92 | 10.65 | 32.00 | 14.00 | 33.89 | 10.34 | 32.00 | 15.00 | 0.001 | 0.81 |
| N index offenses | 2.62 | 2.96 | 2.00 | 2.00 | 2.38 | 3.09 | 1.00 | 3.00 | 0.08 | 0.0005 |
| Length of prison term [months] | 4.59 | 5.80 | 2.86 | 3.89 | 6.51 | 9.27 | 3.23 | 6.14 | 0.30 | 0.0005 |
| Year of admission (first term) | 2010.22 | 3.30 | 2011.00 | 6.00 | 2008.39 | 4.54 | 2008.00 | 9.00 | 0.51 | 0.0005 |
|  |  |  |  |  |  |  |  |  |  |  |
|  | % |  |  |  | % |  |  |  |  |  |
| HAT | 5.77 |  |  |  | 3.15 |  |  |  |  | 0.046 |
| Used psychiatric services | 2.50 |  |  |  | 11.70 |  |  |  |  | 0.0005 |

^a^Analysis on the level of prison terms (Total N = 2279)

^b^Cohen's d

^c^Kruskal-Wallist test for continuous variables, Chi-squared test for frequencies

HAT = Heroin-assisted treatment , IQR = interquartile range, N = Number, SD = Standard deviation

**eTable 4. Effects of missing data on index offense on outcomes and predictors**

|  | Index offense non-missing (N=2118^a^) | | | | Index offense missing (N=161^a^) | | | |  |  |
| --- | --- | --- | --- | --- | --- | --- | --- | --- | --- | --- |
|  | Mean | SD | Median | IQR | Mean | SD | Median | IQR | Effect size^b^ | P-value^c^ |
|  |  |  |  |  |  |  |  |  |  |  |
| N work days | 16.31 | 7.02 | 18.82 | 6.77 | 8.09 | 8.21 | 6.00 | 15.91 | 1.11 | 0.0005 |
| N sick days | 1.52 | 3.73 | 0.00 | 1.01 | 1.24 | 3.27 | 0.00 | 0.47 | 0.07 | 0.066 |
| N weekend days at work | 0.89 | 2.02 | 0000 | 0.21 | 0.29 | 1.20 | 0.00 | 0.00 | 0.30 | 0.001 |
| N weekend days at work | 2.02 | 5.87 | 0.00 | 0.00 | 0.71 | 3.30 | 0.00 | 0.00 | 0.23 | 0.098 |
| Salary | 35.46 | 22.64 | 33.64 | 13.73 | 20.67 | 27.41 | 15.22 | 32.97 | 0.63 | 0.0005 |
| Age | 33.78 | 10.53 | 32.00 | 14.00 | 35.66 | 11.35 | 34.00 | 15.00 | 0.18 | 0.037 |
| Length of prison term [months] | 4.6 | 5.71 | 2.86 | 3.94 | 8.73 | 12.48 | 3.58 | 9.13 | 0.63 | 0.0005 |
| Year of admission (first term) | 2010.26 | 3.35 | 2011.00 | 6.00 | 2005.64 | 3.76 | 2004.00 | 4.00 | 1.29 | 0.0005 |
|  |  |  |  |  |  |  |  |  |  |  |
|  | % |  |  |  | % |  |  |  |  |  |
| HAT | 5.61 |  |  |  | 1.97 |  |  |  |  | 0.055 |
| Used psychiatric services | 4.11 |  |  |  | 1.86 |  |  |  |  | 0.159 |
| Swiss nationality | 36.82 |  |  |  | 36.99 |  |  |  |  | 0.976 |

^a^Analysis on the level of prison terms (Total N = 2279)

^b^Cohen's d

^c^Kruskal-Wallist test for continuous variables, Chi-squared test for frequencies

HAT = Heroin-assisted treatment , IQR = interquartile range, N = Number, SD = Standard deviation

**Power analysis**

We computed power analyses to determine what effects sizes could be detected with 80% power, given our group sizes. In the case of Pearson's Chi-squared test, a module for power analysis is available in Stata. However, to our knowledge, there is no software package in Stata or R to compute power of the Kruskal-Wallis test. We therefore established the relevant parameters for our study by simulation. Many of our variables seem roughly Poisson-distributed, while only a few seem normally distributed (e.g. monthly salary). We repeatedly generated pairs of variables from both distributions with means and variances in the range of our actual variables, and with mean differences (effect sizes) in the range of our actually found group differences (HAT vs Non-HAT). We recorded p-values from the Krukal-Wallis test for each comparison and established statistical power as the proportion of statistically significant test among all tests. Our target were those effect sizes that could be detected with 80% power.

Results:

The size of effects detectable by a Pearson's Chi-squared test comparing frequencies depends on the frequency in the Non-HAT group. We found that for Non-HAT group frequencies between 10% and 90%, which are typical for the present study, effect sizes (differences in percentages) have to be between 10% and 15% to be detectable with a power of 0.8 given present group sizes. In other words, group differences in frequency have to be at least 10 %- 15% to be detectable as statistically significant with 80% probability. This is the case for about two thirds of the effects found in our study.

For Poisson-distributed variables, we found the effect size detectable by a Kruskal-Wallis test to depend on the mean. For means of 0.5 or higher, an effect had to be at least around 0.4 (Cohen's d) to be detectable as statistically significant with 80% power. For means of 0.25 and below, effect size had to be around 0.5 and 0.6, respectively. For normally distributed variables, the detectable effect size was 0.3. Overall, therefore, the test is moderately sensitive under the given conditions.
